# Tyrosine Phosphorylation of SGEF Regulates RhoG Activity and Cell Migration

**DOI:** 10.1371/journal.pone.0159617

**Published:** 2016-07-20

**Authors:** Yusuke Okuyama, Kentaro Umeda, Manabu Negishi, Hironori Katoh

**Affiliations:** 1 Laboratory of Molecular Neurobiology, Graduate School of Pharmaceutical Sciences, Kyoto University, Yoshidakonoe-cho, Sakyo-ku, Kyoto 606-8501, Japan; 2 Laboratory of Molecular Neurobiology, Graduate School of Biostudies, Kyoto University, Yoshidakonoe-cho, Sakyo-ku, Kyoto 606-8501, Japan; Hungarian Academy of Sciences, HUNGARY

## Abstract

SGEF and Ephexin4 are members of the Ephexin subfamily of RhoGEFs that specifically activate the small GTPase RhoG. It is reported that Ephexin1 and Ephexin5, two well-characterized Ephexin subfamily RhoGEFs, are tyrosine-phosphorylated by Src, and that their phosphorylation affect their activities and functions. In this study, we show that SGEF, but not Ephexin4, is tyrosine-phosphorylated by Src. Tyrosine phosphorylation of SGEF suppresses its interaction with RhoG, the elevation of RhoG activity, and SGEF-mediated promotion of cell migration. We identified tyrosine 530 (Y530), which is located within the Dbl homology domain, as a major phosphorylation site of SGEF by Src, and Y530F mutation blocked the inhibitory effect of Src on SGEF. Taken together, these results suggest that the activity of SGEF is negatively regulated by tyrosine phosphorylation of the DH domain.

## Introduction

It is already well known that members of the Rho family of small GTPases play pivotal roles in the regulation of cell morphology and migration. These cellular functions contribute to many steps in cancer initiation and progression [1–3]. Like other small GTPases, Rho family GTPases serve as molecular switches by cycling between an inactive GDP-bound state and an active GTP-bound state, and activated GTPases can bind to their specific effectors that lead to a variety of biological functions. Activation of the Rho family proteins requires GDP-GTP exchange catalyzed by various guanine nucleotide exchange factors (GEFs), whereas the activation of the GTPases is down-regulated by GTPase-activating proteins, which stimulate the intrinsic GTPase activities.

RhoG is a member of Rho family small GTPases that is a key upstream regulator of another Rho family member Rac, and induces diverse cellular functions, including promotion of cell migration, neurite outgrowth in neuronal cells, and stimulation of phagocytosis [4–7]. ELMO, an effector for RhoG, forms a complex with Rac GEF Dock180 or Dock4, and when RhoG is activated, it binds to ELMO to induce translocation of the ELMO-Dock180 or ELMO-Dock4 complex from the cytoplasm to the plasma membrane, leading to activation of Rac [6,8,9]. On the other hand, RhoG binds to phosphatidylinositol 3-kinase (PI3K) p85α regulatory subunit and activates the PI3K/Akt signaling pathway to promote cell proliferation and survival independently of the activation of Rac [10–12].

SGEF and Ephexin4 (also known as ARHGEF16) are closely related Dbl type RhoGEFs that specifically activates RhoG [13, 14]. Ephexin4 interacts with a tyrosine kinase receptor EphA2 and mediates ligand ephrin-independent promotion of cell migration and suppression of anoikis through activation of RhoG [14–17]. Ephexin4-mediated RhoG activation is also involved in engulfment of apoptotic cells and epithelial morphogenesis [18,19]. SGEF contributes to the formation of actin rich protrusions on the dorsal surface of endothelial cells, and promotes leukocyte trans-endothelial migration and blood vessel lumen morphogenesis [20,21]. SGEF is also involved in EGF receptor stability and signaling [22,23], remodeling of actin cytoskeleton stimulated by *Salmonella* [24], and formation of atherosclerosis [25]. On the other hand, SGEF is overexpressed in several types of cancers and promotes cancer cell growth and migration [26,27]. However, it is not fully understood how the activities of SGEF and Ephexin4 are regulated. In this study, we show that SGEF, but not Ephexin4, is tyrosine-phosphorylated by Src on tyrosine 530 (Y530), which is located within the Dbl homology (DH) domain, leading to suppression of SGEF-RhoG interaction and SGEF-mediated cell migration.

## Materials and Methods

### Plasmids and antibodies

The expression plasmid pCAG encoding YFP and pCXN2 vector [28] were generous gifts from Dr J. Miyazaki (Osaka University, Osaka, Japan). Src-Y527F was from Drs. T. Akagi (KAN Research Institute, Kobe, Japan) and M. Matsuda (Kyoto University, Kyoto, Japan) and subcloned into pEF-BOS with a HA tag sequence at the N-terminus. Plasmids expressing Flag-tagged Ephexin4, Myc-tagged RhoG, and GST-fused ELMO-NT were obtained as described previously [4,8,14]. Mouse SGEF sequence was amplified by RT-PCR from mouse brain RNA and subcloned into pCXN2 with a Flag tag sequence at the N-terminus. SGEF-Y378F, -Y452F, -Y527F, and -Y530F, and RhoG-G15A were generated by PCR-mediated mutagenesis and subcloned into pCXN2 or pGEX4T-2.

The following antibodies were used in this study: a mouse monoclonal antibody (mAb) against Myc (9E10, Santa Cruz Biotechnology); a mouse mAb against Flag (M2, Sigma); a mouse mAb against HA (3F10, Roche); a mouse mAb against phosphotyrosine (4G10, Millipore); secondary antibodies conjugated to horseradish peroxidase (DAKO).

### Cell culture and transfection

HEK293T cells were grown in Dulbecco’s modified Eagle’s medium (DMEM) containing 10% FBS, 4 mM glutamine, 100 units/ml of penicillin, and 0.1 mg/ml of streptomycin under humidified air containing 5% CO_2_ at 37°C. Cells were transfected with the indicated plasmids using LipofectAMINE Plus, LipofectAMINE 2000 (Invitrogen), or polyethyleneimine MAX, according to the manufacturer’s instructions.

### Transwell cell migration assay

Transwell cell migration assay was performed as described previously [14, 16]. HEK293T cells were transfected with YFP alone, or together with the indicated plasmids and incubated for 24 h. The cells were detached and then resuspended in serum free DMEM. The cells were replated onto the upper chamber of a Transwell filter (Costar, 8 μm pore size). DMEM supplemented with 10% FBS was added to the lower chamber. At 6 h after plating, cells were fixed with 4% paraformaldehyde in phosphate buffered saline. Non-migrated cells on the upper side of the filter were removed with a cotton swab. In parallel, cells were separately plated to culture wells without the Transwell filters for estimating the total number of attached cells. Cell migration was calculated by the number of YFP-positive cells underside of the filter normalized to the total number of the YFP-positive attached cells. For each experiment, the number of cells in at least 8 random fields on the underside of the filter was counted, and four or five independent filters were analyzed.

### Immunoprecipitation and immunoblotting

Immunoprecipitation and immunoblotting were performed as described in detail previously [15, 17, 29]. After transfection, HEK293T cells were lysed for 10 min with ice-cold cell lysis buffer (20 mM Tris-HCl, pH7.5, 150 mM NaCl, 2 mM MgCl_2_, 1% TritonX-100, 10 mM NaF, 1 mM Na_3_VO_4_, 1 mM PMSF, 10 μg/ml Aprotinin, 10 μg/ml Leupeptin). After centrifugation for 10 min at 16,000 x *g*, the supernatants were incubated with anti-Flag antibody for 1 h followed by incubation with protein G Sepharose (GE Healthcare) for 1 h. After the beads were washed with the cell lysis buffer, the bound proteins were eluted in Laemmli sample buffer. The eluted proteins were separated by SDS-PAGE, and were electrophoretically transferred onto a polyvinylidene difluoride membrane (Millipore Corporation). The membrane was blocked with 3% low fat milk in Tris-buffered saline, and then incubated with primary antibodies. The primary antibodies were detected with horseradish peroxidase-conjugated secondary antibodies and chemiluminescence detection kit (ECL Prime, GE Healthcare; Chemi-Lumi One, Nacalai Tesque).

### Measurement of RhoG activity

Measurement of RhoG activity in cells was performed as described previously [8, 15, 17]. The N-terminal RhoG-binding domain of ELMO2 (ELMO-NT, amino acids 1–362) was expressed in *Escherichia coli* as a fusion protein with GST and purified on glutathione-Sepharose beads (GE Healthcare). Protein concentration was determined by comparing with bovine serum albumin standards after SDS-PAGE and by staining with Coomassie Brilliant Blue. To determine RhoG activity in HEK293T cells, transfected cells were lysed with the ice-cold cell lysis buffer (10 mM Tris-HCl, pH 7.5, 100 mM NaCl, 2 mM MgCl2, 1% Nonidet P-40, 10% glycerol, 1 mM DTT, 1 mM PMSF, 10 μg/ml aprotinin, and 10 μg/ml leupeptin). Cell lysates were then centrifuged for 5 min at 10,000 x *g* at 4°C, and the supernatant was incubated with 20 μg of GST-ELMO-NT bound to glutathione-Sepharose beads for 30 min at 4°C. The beads were washed with lysis buffer, and bound proteins were analyzed by SDS-PAGE and immunoblotting.

### Pull-down assay

GST-RhoG-G15A was expressed in *Escherichia coli* and purified on glutathione-Sepharose beads. HEK293T cells transfected with Flag-SGEF were lysed for 10 min with ice-cold cell lysis buffer (20 mM Tris-HCl, pH7.5, 150 mM NaCl, 2 mM MgCl_2_, 10 mM NaF, 1 mM Na_3_VO_4_, 1% Triton X-100, 1 mM PMSF, 10 μg/ml aprotinin, 10 μg/ml leupeptin). After centrifugation for 10 min at 16,000 x *g*, the supernatants were incubated with GST-fused RhoG-G15A bound to glutathione-Sepharose beads for 1 h. The beads were washed with the ice-cold cell lysis buffer, and bound proteins were analyzed by SDS-PAGE and immunoblotting.

## Results

### SGEF, but not Ephexin4, is-tyrosine phosphorylated by Src

**Fig 1:**
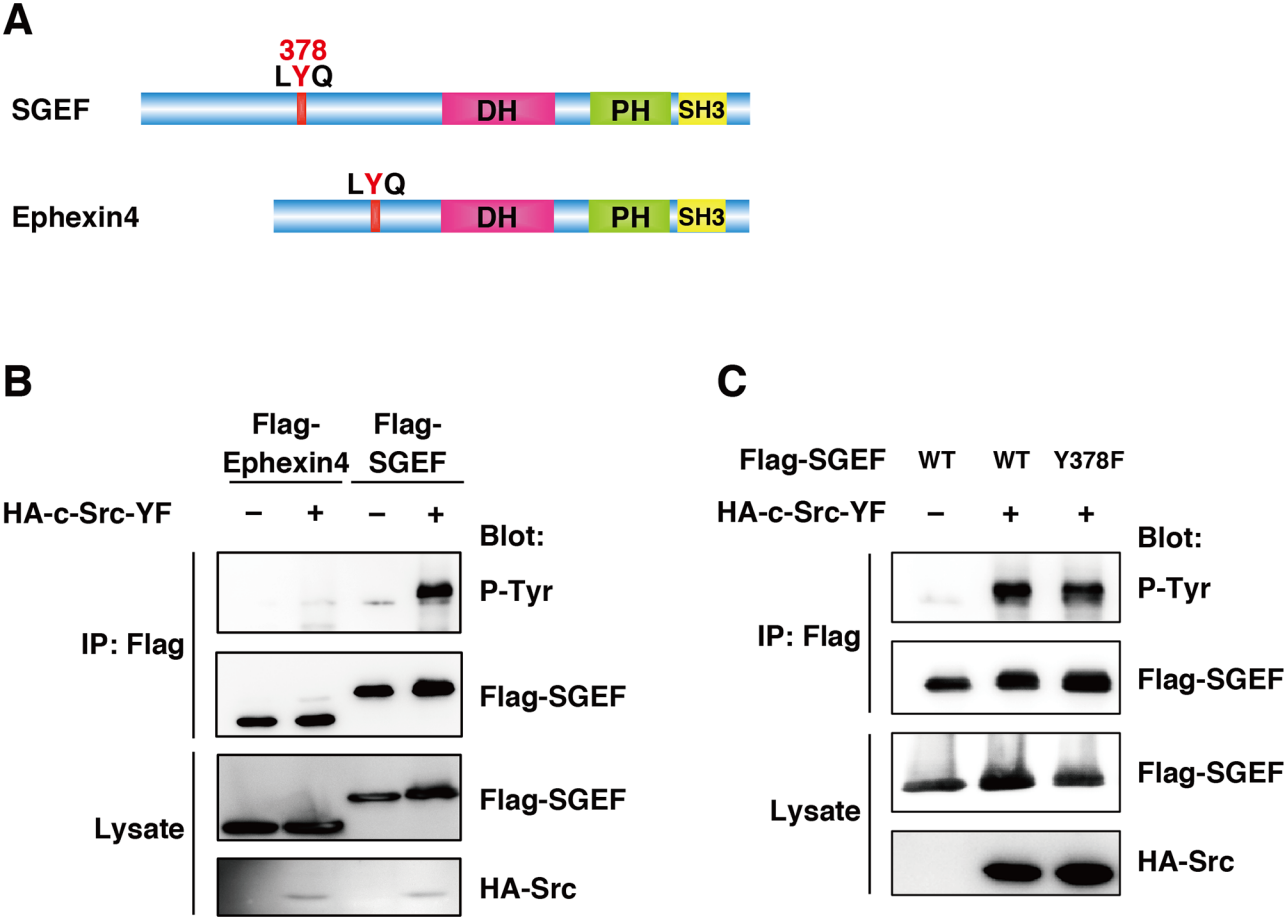
SGEF is tyrosine-phosphorylated by Src. (A) The domain structure of SGEF and Ephexin4. Tyrosine residues in the LYQ motif correspond to the phosphorylation sites of Ephexin1 and Eohexin5 by Src. (B, C) Cell lysates from HEK293T cells transfected with the indicated plasmids were immunoprecipitated with anti-Flag antibody, and tyrosine phosphorylation (p-Tyr) and total cell lysates were analyzed by immunoblotting with antibodies against phospho-tyrosine, Flag, and HA.

SGEF and Ephexin4 are closely related Dbl type RhoGEFs, both of which specifically activate RhoG (Fig 1A) [13,14]. They are also considered as the members of a subfamily of Ephexin [30,31]. Among the Ephexin subfamily members, Ephexin1, Ephexin5, and ARHGEF5/Tim, which are GEFs for RhoA, are tyrosine-phosphorylated by Src or Eph receptors, and their phosphorylation is important for the GEF activity or protein stability [30,32–34]. To examine whether tyrosine phosphorylation also regulates the activities of RhoG-specific GEFs SGEF and Ephexin4, HEK293T cells were co-transfected with HA-tagged constitutively active Src, Src-Y527F (Src-YF) and Flag-tagged SGEF or Ephexin4. Then they were immunoprecipitated from the cell lysates with anti-Flag antibody and immunoblotted with anti-phosphotyrosine antibody 4G10. We found that SGEF was tyrosine phosphorylated in the presence of Src-YF, whereas we can not detect a clear band of phosphorylated Ephexin4 (Fig 1B). Previous studies have shown that Ephexin1 and Ephexin5 are tyrosine phosphorylated in a conserved N-terminal motif (LYQ) among Ephexin subfamily members [30,32]. SGEF also contains an analogous tyrosine residue in the N-terminus (Y378, Fig 1A), raising the possibility that Y378 might be a phosphorylation site of SGEF by Src. However, a mutant of SGEF in which Y378 is substituted with a phenylalanine (SGEF-Y378F) was tyrosine-phosphorylated when it was co-transfected with Src-YF to a level comparable with that of wild-type SGEF (Fig 1C). These results suggest that SGEF, but not Ephexin4, is tyrosine-phosphorylated by Src in a region other than the conserved N-terminal LYQ motif.

### Src suppresses SGEF-mediated RhoG activation and promotion of cell migration

**Fig 2:**
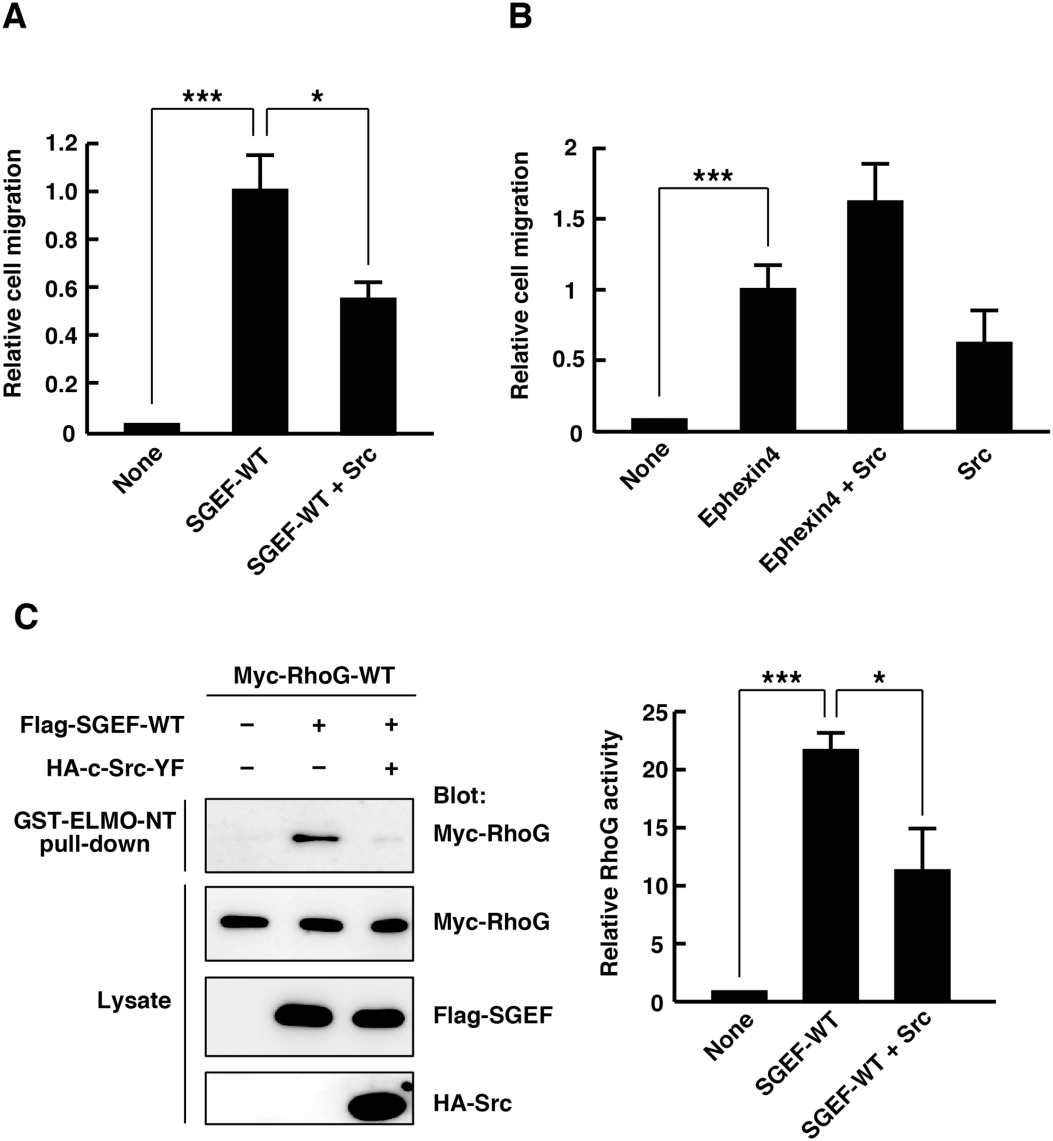
Expression of constitutively active Src suppresses SGEF-mediated promotion of cell migration and RhoG activation. (A) Motility of HEK293T cells was evaluated by Transwell cell migration assays. HEK293T cells transfected with YFP alone or together with the indicated plasmids were plated in the upper chamber of the filters, and cells that had migrated to the underside of the filters were analyzed at 6 h after plating. Results are expressed as means ± SEM (n = 5) of relative cell migration, with SGEF-WT-transfected cell migration set at 1 (*P < 0.05, ***P < 0.001, *t*-test). (B) HEK293T cells transfected with YFP alone or together with the indicated plasmids were plated in the upper chamber of the filters, and cells that had migrated to the underside of the filters were analyzed at 6 h after plating. Results are expressed as means ± SEM (n = 4) of relative cell migration, with Ephexin4-WT-transfected cell migration set at 1 (ns, not significant, ***P < 0.001, *t*-test). (C) Cell lysates from HEK293T cells transfected with indicated plasmids were incubated with GST-ELMO-NT, and bound Myc-RhoG (GST-ELMO-NT pull-down) and total cell lysates were analyzed with antibodies against Myc, Flag, and HA. The amount of bound Myc-RhoG was normalized to the total amount of Myc RhoG in cell lysates, and relative RhoG activity was expressed as fold change relative to the basal activity (None). Data are presented as means ± SEM from four independent experiments (*P < 0.05, ***P < 0.001, *t*-test).

Previous studies reported that SGEF and Ephexin4 mediate promotion of cell migration [14,16,27]. Therefore, we next investigated the effect of Src-YF expression on the SGEF- and Ephexin4-mediated promotion of cell migration. Using an in vitro transwell migration assay, overxpression of wild-type SGEF (SGEF-WT) or Ephexin4 in HEK293T cells promoted cell migration. Co-expression of Src-YF with SGEF-WT significantly suppressed the SGEF-mediated promotion of cell migration (Fig 2A). In contrast, expression of Src-YF did not suppress the Ephexin4-mediated promotion of cell migration (Fig 2B). These results raised the possibility that phosphorylation of SGEF by Src affects SGEF activity. To explore this possibility, we measured RhoG activity in cells expressing SGEF-WT alone or together with Src-YF by a pull-down assay with GST-fused N-terminal RhoG-binding region of ELMO (GST-ELMO-NT), which could specifically interact with GTP-bound active RhoG [8,14]. Expression of SGEF-WT in HEK293T cells increased the amount of active RhoG, while co-expression of Src-YF significantly suppressed the SGEF-induced RhoG activation (Fig 2C). These results suggest that Src acts as a negative regulator of SGEF.

### Phosphorylation of SGEF by Src suppresses the interaction between SGEF and RhoG

**Fig 3:**
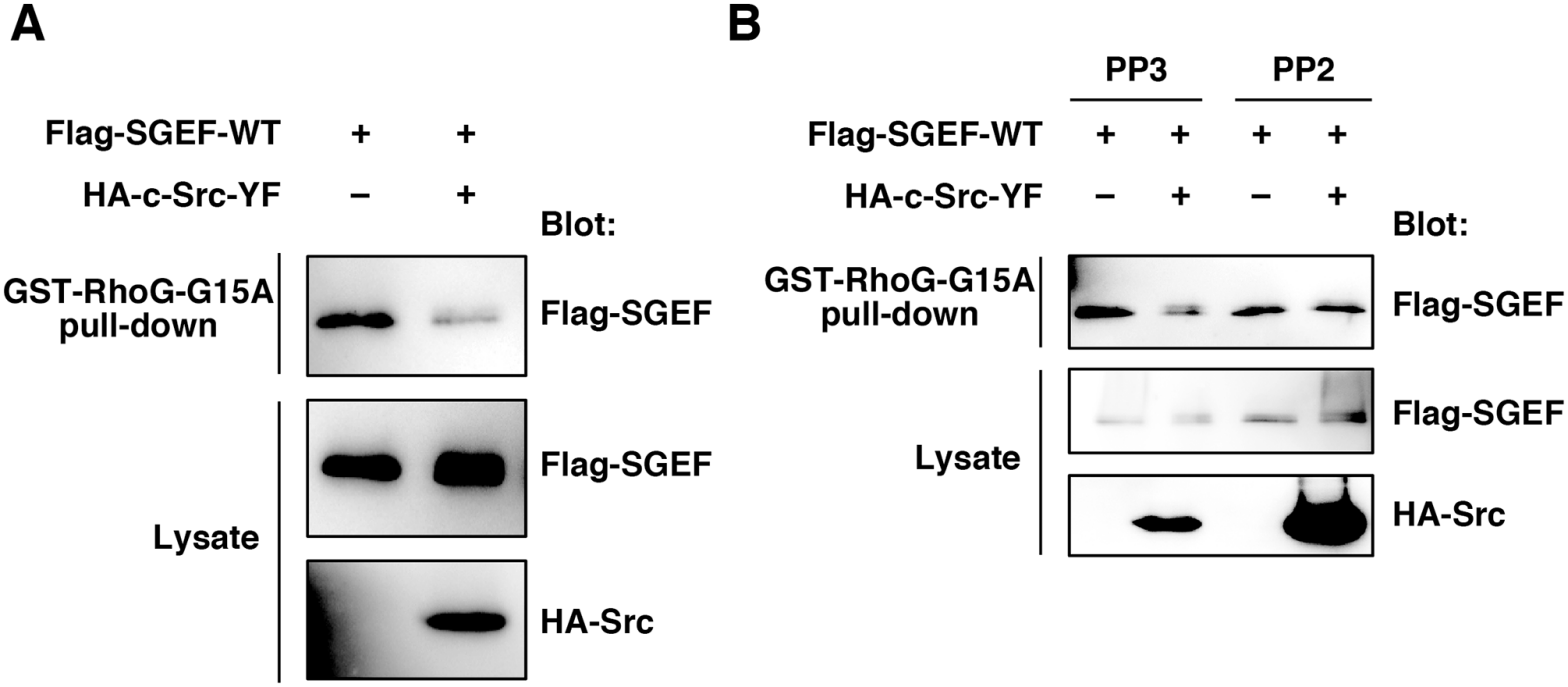
Phosphorylation of SGEF by Src blocks the interaction of SGEF with RhoG. (A) Cell lysates from HEK293T cells transfected with the indicated plasmids were incubated with GST-RhoG-G15A, and bound Flag-SGEF (GST-RhoG-G15A pull-down) and total cell lysates were analyzed by immunoblotting with anti-Flag and anti-HA antibodies. (B) HEK293T cells were transfected with the indicated plasmids and treated for 30 min with 20 μM PP2 or PP3. Then cell lysates were incubated with GST-RhoG-G15A, and bound Flag-SGEF and total cell lysates were analyzed by immunoblotting with anti-Flag and anti-HA antibodies.

To elucidate the mechanism of regulation of SGEF activity by Src, we examined the interaction between SGEF and RhoG. HEK293T cells were transfected with Flag-tagged SGEF-WT alone or together with HA-tagged Src-YF, and the cell lysates were used in a pull-down assay with purified GST-fused RhoG containing the G15A mutation (RhoG-G15A), which binds with high affinity to SGEF [27]. SGEF bound to GST-fused RhoG-G15A, but co-expression of Src-YF inhibited the SGEF-RhoG interaction (Fig 3A). To confirm that the kinase activity of Src is required for its inhibitory effect on the SGEF-RhoG interaction, HEK293T cells transfected with Flag-SGEF-WT and HA-Src-YF were treated with the Src family kinase inhibitor PP2 or its inactive analogue PP3, and the cell lysates were used in GST pull-down assay. PP2 treatment abolished Src-YF-mediated inhibition of the SGEF-RhoG interaction, whereas PP3 treatment had no effect (Fig 3B). These results suggest that phosphorylation of SGEF by Src inhibits SGEF-RhoG interaction.

### Y530 is identified as an important phosphorylation site in SGEF

**Fig 4:**
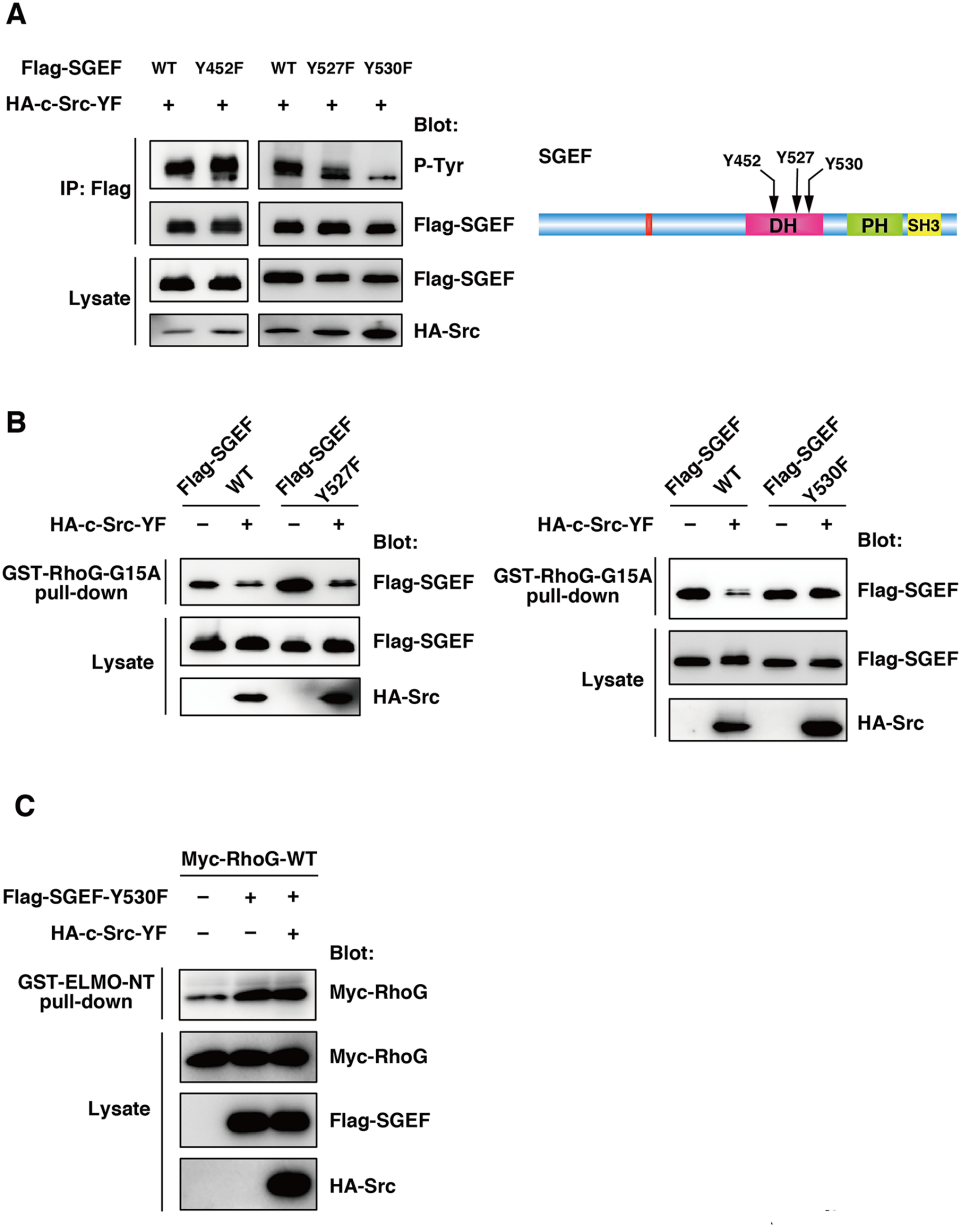
Y530 is an important phosphorylation site in SGEF. (A) Cell lysates from HEK293T cells transfected with the indicated plasmids were immunoprecipitated with anti-Flag antibody, and tyrosine phosphorylation (p-Tyr) and total cell lysates were analyzed by immunoblotting with antibodies against phospho-tyrosine, Flag, and HA. (B) Cell lysates from HEK293T cells transfected with the indicated plasmids were incubated with GST-RhoG-G15A, and bound Flag-SGEF and total cell lysates were analyzed by immunoblotting with anti-Flag and anti-HA antibodies. (C) Cell lysates from HEK293T cells transfected with indicated plasmids were incubated with GST-ELMO-NT, and bound Myc-RhoG and total cell lysates were analyzed with antibodies against Myc, Flag, and HA.

Because tyrosine phosphorylation of SGEF inhibited its interaction with RhoG, we speculated that a tyrosine residue(s) within the DH domain are an important phosphorylation site(s) by Src. There are five tyrosine residues in the DH domain of SGEF. Among them, Y452, Y527, and Y530 of SGEF are candidate tyrosine residues, because their corresponding amino acids in RacGEF Tiam1 are located near the Tiam1/Rac1 interface in the crystal structure of the DH domain of Tiam1 in complex with Rac1 [35]. In addition, these tyrosine residues are conserved in five mammalian SGEFs and the chicken and Xenopus SGEF orthologs. We therefore generated three SGEF mutants (SGEF-Y452F, -Y527F, -Y530F) to examine whether Src-YF can phosphorylate these SGEF mutants. HEK293T cells were co-transfected with HA-tagged Src-YF and Flag-tagged SGEF-WT or SGEF mutants. Then they were immunoprecipitated from the cell lysates with anti-Flag antibody and immunoblotted with anti-phosphotyrosine antibody. SGEF-Y452F and -Y527F were tyrosine-phosphorylated by Src-YF to a level similar to SGEF-WT. However, Y530F mutation greatly reduced Src-YF-induced SGEF tyrosine phosphorylation (Fig 4A), indicating that Y530 is one of major phosphorylation site of SGEF by Src. To test whether phosphorylation of Y530 regulates the interaction between SGEF and RhoG, HEK293T cells were transfected with Flag-tagged SGEF alone or together with HA-tagged Src-YF, and the cell lysates were used in a pull-down assay with GST-RhoG-G15A. Co-exoression of Src-YF with SGEF-WT or -Y527F mutant inhibited their interaction with RhoG-G15A. However, co-expression of Src-YF had little effect on the interaction between SGEF-Y530F and RhoG-G15A (Fig 4B). Co-expression of Src-YF also had little effect on the SGEF-Y530F-induced RhoG activation (Fig 4C). These results suggest that Y530 phosphorylation of SGEF negatively regulates SGEF-RhoG interaction and the RhoG activation.

**Fig 5:**
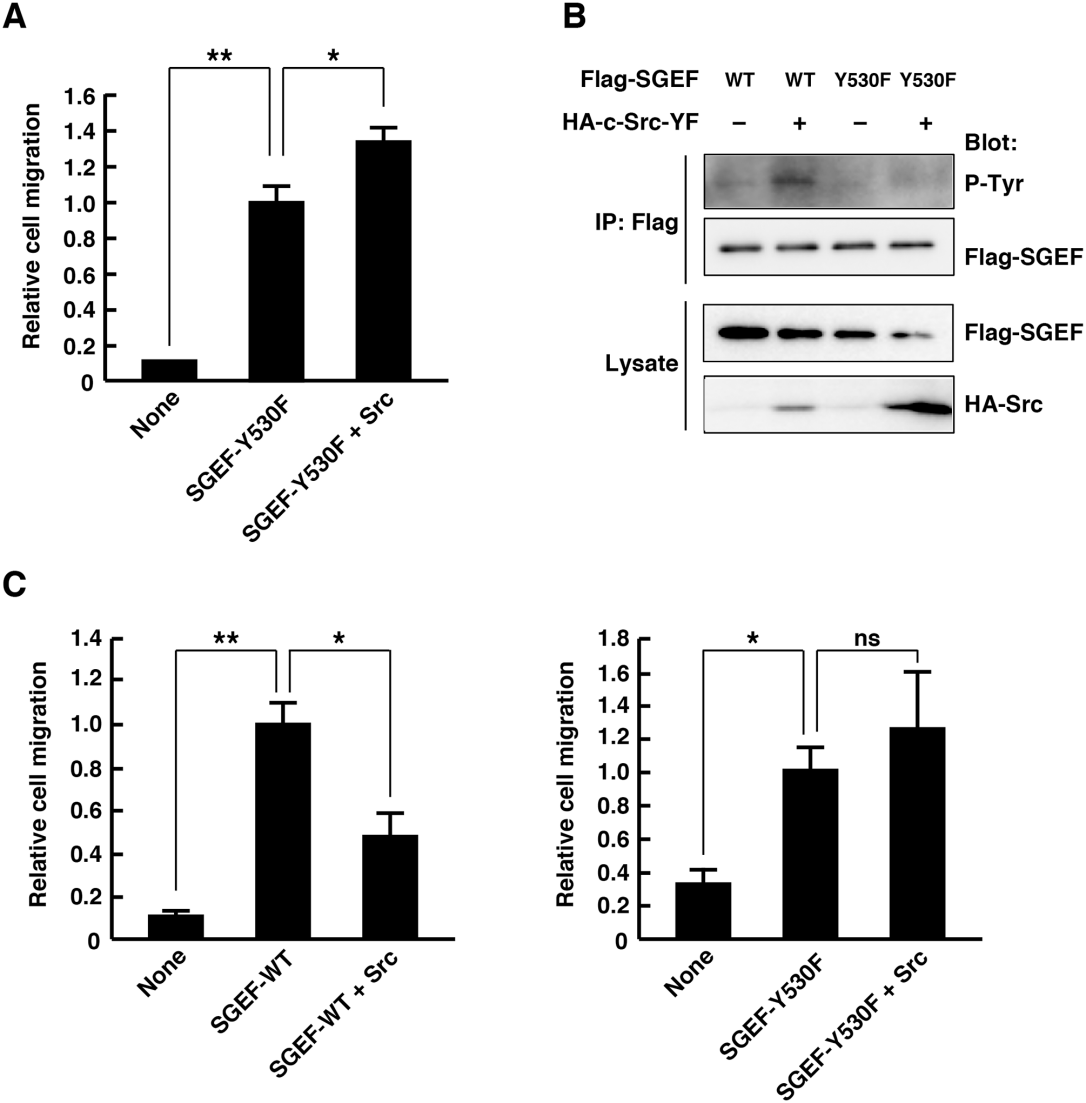
Src does not block the SGEF-Y530F-mediated promotion of cell migration. (A) HEK293T cells transfected with YFP alone or together with the indicated plasmids were plated in the upper chamber of the filters, and cells that had migrated to the underside of the filters were analyzed at 6 h after plating. Results are expressed as means ± SEM (n = 3) of relative cell migration, with SGEF-Y530F-transfected cell migration set at 1 (*P < 0.05, **P < 0.01, *t*-test). (B) Cell lysates from HeLa cells transfected with the indicated plasmids were immunoprecipitated with anti-Flag antibody, and tyrosine phosphorylation (p-Tyr) and total cell lysates were analyzed by immunoblotting with antibodies against phospho-tyrosine, Flag, and HA. (C) HeLa cells transfected with YFP alone or together with the indicated plasmids were plated in the upper chamber of the filters, and cells that had migrated to the underside of the filters were analyzed at 6 h after plating. Results are expressed as means ± SEM (n = 3) of relative cell migration, with SGEF-WT or Y530F-transfected cell migration set at 1 (ns, not significant, *P < 0.05, **P < 0.01, *t*-test).

We next tested whether Y530 phosphorylation of SGEF regulates SGEF-mediated promotion of cell migration. Although co-expression of Src-YF with SGEF-WT significantly suppressed the SGEF-mediated promotion of cell migration (Fig 2A), expression of Src-YF had little effect on the SGEF-Y530F-mediated promotion of cell migration (Fig 5A). These results suggest that Y530F phosphorylation of SGEF plays an important role in the regulation of SGEF activity and function. To examine whether the regulation of SGEF by tyrosine phosphorylation is cell type dependent, we used another cell line HeLa. Consistent with the results obtained from HEK293T cells, expression of Src-YF induced tyrosine phosphorylation of SGEF-WT, but not SGEF-Y530F, in HeLa cells (Fig 5B). In addition, Src-YF significantly suppressed the SGEF-WT-induced promotion of cell migration, whereas it had little effect on the SGEF-Y530F-mediated promotion of cell migration (Fig 5C). Thus, the regulation of SGEF by tyrosine phosphorylation is not specific to HEK293T cells.

## Discussion

Small GTPase RhoG is a key upstream regulator of small GTPase Rac through its effector ELMO/Dock180 or ELMO/Dock4 complex [8,9]. Activation of Rac induces the formation of actin-rich lamellipodia protrusions, which serves as a major driving force of cell movement, and Rac activation is essential for cell migration in various cell types [36, 37]. Therefore, the regulation of RhoG activity is important for the control of Rac activity and cell motility. In fact, abnormal RhoG activation leads to promotion of cancer cell migration and invasion [14,27,38]. SGEF is a RhoG-specific GEF, and also promotes cell migration and invasion in glioblastoma [27]. SGEF-mediated RhoG activation occurs in response to some stimuli [20,27], but its regulatory mechanism is not well understood. In this study, we provide evidence that the GEF activity of SGEF is regulated by its tyrosine phosphorylation. Tyrosine kinase Src phosphorylates SGEF on Y530, which is located within the DH domain. SGEF Y530 phosphorylation blocks its interaction with RhoG and suppresses RhoG activation, leading to reduced cell migration. Thus, this is a novel mechanism regulating RhoG activity and cell motility, and may contribute to preventing over-activation of RhoG and aberrant cell migration.

The domain structure of SGEF is similar to Ephexin subfamily RhoGEFs: they contain the DH-PH domain followed by the SH3 domain at the C-terminus. SGEF also has a conserved motif among Ephexin subfamily members at the N-terminus, LYQ, and the tyrosine residues in this motif of Ephexin1 and Ephexin5 are phosphorylated by Src or Eph receptors [30,32]. Tyrosine phosphorylation of Ephexin1 in the LYQ motif promotes its GEF activity against RhoA, whereas tyrosine phosphorylation of Ephexin5 triggers its ubiquitination and degradation. SGEF and Ephexin4 are RhoG-specific GEFs among the Ephexin subfamily members, and also have the LYQ motif at the N-terminus. However, our results reveal that SGEF is not phosphorylated on the tyrosine residue in this motif but phosphorylated on Y530 within the DH domain. On the other hand, Src does not appear to induce tyrosine phosphorylation of Ephexin4. Src phosphorylates another Ephexin member Tim (ARHGEF5) on two tyrosine residues in the N-terminus, and phosphorylation of these tyrosine residues promotes its GEF activity by relieving the autoinhibitory intramolecular interaction [33]. Thus, the regulatory mechanisms of the GEF activity by tyrosine phosphorylation are different among Ephexin subfamily members.

Phosphorylation of RhoGEFs is one of major regulatory mechanisms for both stimulation and inhibition of their activities. It is well known that the activity of Vav is positively regulated by tyrosine phosphorylation, which releases the autoinhibitory intramolecular interaction between the N-terminal region and the DH domain [39]. Phosphorylation-dependent stimulation of the GEF activity is also observed in several RhoGEFs, including Tiam1 [40], GEF-H1 [41], Dbs [42], and FLJ00018/PLEKHG2 [43]. On the other hand, some RhoGEFs are inhibited by their phosphorylation. For example, Aurora A/B and Cdk1/Cyclin B phosphorylate GEF-H1 and inhibit the GEF activity [44]. Protein kinase A phosphorylates Lfc and β_1_Pix and promotes their interactions with 14-3-3, leading to inhibition of their GEF activities [45,46]. However, phosphorylation sites of these RhoGEFs are not located within the DH domain, and little is known about the regulation of RhoGEF activity by phosphorylation of the DH domain. Gupta et al. recently reported that Dbl, a GEF for RhoA and Cdc42, is phosphorylated on a tyrosine residue within the DH domain, leading to its interaction with Grb2 and promotion of the GEF activity [47]. Our results demonstrate that tyrosine phosphorylation within the DH domain of SGEF inhibits its interaction with RhoG and RhoG activation. However, there is still one major question whether phosphorylation of SGEF directly inhibits its ability to bind RhoG, because our data does not rule out the possibility that other proteins in the cell lysate may bind phosphorylated SGEF and prevent the interaction with, and subsequent activation of RhoG. We will address this issue in future work.
